# Researching Sensitive Topics: The Value of Inclusive Patient and Public Involvement and Engagement in the Design and Implementation of the Larger Bodies in Radiography Project

**DOI:** 10.1111/hex.70563

**Published:** 2026-02-08

**Authors:** Hancock Amy, Heales Christine, Ulett Poppy, Graham Carolyn, Manning Fay

**Affiliations:** ^1^ Department of Health and Care Professions. South Cloisters University of Exeter Exeter UK; ^2^ Patient Representative Exeter UK

**Keywords:** access barriers, challenging topics, inclusion, medical imaging, radiotherapy, representation, sensitivities

## Abstract

**Introduction:**

Weight stigma and bias in healthcare are well‐documented and can deter individuals from participating in research. These sensitivities often foster distrust, creating barriers to recruitment and engagement. Patient and Public Involvement and Engagement (PPIE) strategies are essential to building trust, enhancing transparency and fostering collaboration with under‐represented populations.

**Methods:**

This article presents both stages of the PPIE strategy for the Larger Bodies in Radiography (LBinRAD), a multiphased research project which aims to explore experiences and develop services which support person‐centred, compassionate care for individuals with larger bodies across medical imaging and radiotherapy services. Stage 1 involved four individuals with lived experience who contributed to developing a UK‐wide survey, disseminated via social media to recruit participants living in larger bodies who had accessed radiographic services. Stage 2 created a pool of 28 PPIE representatives, inviting them to participate in five online focus groups designed to: (i) explore participants' perspectives on key priorities, including widening participation; (ii) co‐create belief statements to guide future PPIE activities; and (iii) collaboratively shape subsequent research phases.

**Results:**

In Stage One, PPIE consultation shaped language and messaging, leading to the adoption of the term ‘larger body′ and clarification that body weight data would not be collected. A distinct sub‐population identifying as taller than average was also recognised. PPIE representatives recommended targeted outreach to relevant social media groups, significantly improving recruitment reach and inclusivity.

In Stage Two, 22 PPIE representatives participated across five focus groups. Listening to their experiences provided valuable insights that helped ensure lived experience remained central to the methodology. These insights informed several key refinements, including broadening inclusion criteria and recruitment locations, exploring language in greater depth, discussing cultural influences, and using varied media and methods to capture data and support co‐design.

**Conclusion:**

PPIE was central to the success of Phase 1, shaping the research process and outcomes. Insights and action points from this collaborative approach will guide Phase 2 and future research development. By centring historically marginalised voices, PPIE enhances research inclusivity, relevance, credibility and impact.

**Patient or Public Contribution:**

The research was undertaken in partnership with PPIE representatives, and this publication has been co‐authored with a representative, who will be a co‐applicant for future research funding.

**Acronyms**
CoRIPS: College of Radiographers Industry Partnership Scheme
GDPR: General Data Protection Regulation
LBinRAD: **L**arger **B**odies **in Rad**iography
NIHR: National Institute for Health Research
PiS: Participant Information Sheet
PPIE: Patient and Public Engagement and Involvement
PER: Public Engagement with Research

## 1 Introduction

The occurrence of patients with a larger body* is becoming more commonplace in medical imaging and radiotherapy services [1, 2, 3, 4]. Therapeutic and diagnostic radiographers can face technical challenges and have reported a lack of preparedness when caring for patients, despite the now modern normalcy of those who would be classified or classify themselves as being part of this population [5]. It has also been shown that some radiographers face communication difficulties with ‘larger′ patients, worrying about appearing as ‘unknowingly offensive′ [5]. Biases against those with a larger body are recognised across healthcare, with many individuals reporting feelings of being judged and blamed [6]. The recent heightening of weight stigma has been attributed to an increase in ‘blame and shame framing′ of obesity in the media and public health [7, 8]. Weight stigma towards these individuals within the healthcare setting has been shown to negatively impact the quality of care and healthcare outcomes [9, 10, 11, 12, 13].

Whilst there is a growing international body of evidence which explores radiographers [14, 15] and wider healthcare professionals' [16, 17, 18] perceptions of caring for patients with a larger body, there is currently a distinct lack of exploration from the patient's perspective, with no such research undertaken to understand their experiences of UK radiotherapy and diagnostic radiography services.

**L**arger **B**odies **in Rad**iography (LBinRAD) is a multiphased research project which aims to explore experiences and develop services which support person‐centred, compassionate care for individuals with larger bodies across medical imaging and radiotherapy services. Issues of weight stigma experienced in healthcare can transcend into healthcare research, subsequently causing larger‐bodied individuals to be suspicious about the researcher's intent. Recruitment and retention for studies can therefore be challenging, with potential participants fearful that any investigation will include disguised attempts to encourage lifestyle change and promote weight loss. Our research needed to be transparent and non‐ambiguous, to not reinforce or validate any existing distrust of healthcare professionals' intentions [19, 20]. Establishing and maintaining trust with our target population was therefore essential if the project was to be successful and to avoid research wastage caused by poor design and delivery [21].

The primary outcome was the development and integration of a Patient and Public Involvement and Engagement (PPIE) strategy, designed to support project inception and remain embedded throughout each stage of the research. This approach aimed to ensure the creation of valid, real‐world findings and their implementation into medical imaging and radiotherapy practice.

**Figure 1:**
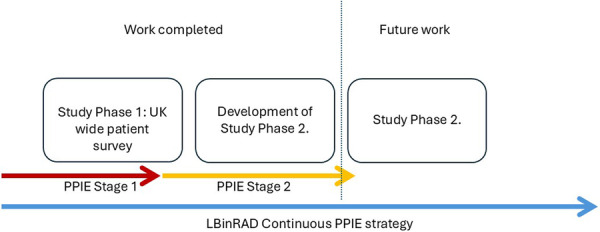
LBinRAD project overview. *Note:* Although this term is used within the introduction, it was not adopted until our PPIE strategy had been employed. The term was recommended for use by our PPIE representatives in Stage 1.

The focus of this article is to report on the first two stages of the overarching LBinRAD PPIE strategy (Figure 1), detailing the processes employed to embed the patient voice into our research whilst outlining the value and benefit of involving and engaging representatives with lived experiences throughout.

## 2 Materials and Methods

### 2.1 PPIE Stage 1

**Figure 2:**
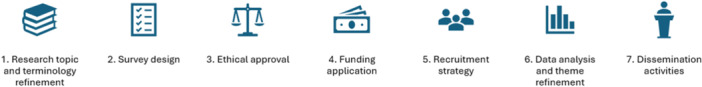
Component research activities of Phase 1.

Prior to the project commencement, the authors sought the involvement of PPIE representatives who self‐identified as living in a larger body. PPIE recruitment was undertaken via social media, reaching out to individuals who were active in the body‐positive sphere and using the research teams' own UK‐wide PPIE networks. The level of engagement amongst representatives was designed to be fluid and offered the opportunity to input and engage with components of the research (Figure 2), whilst understanding they were under no obligation to commit. Whilst it is recommended that a minimum of two representatives sit on steering groups [22], the ‘right number′ of people to engage should ultimately be guided by the study [23]. Therefore, to ensure there was some range of diversity of backgrounds and experiences and to build capacity, it was decided a minimum of four representatives would be recruited. An honorarium payment was offered to the representatives guided by the National Institute for Health and Care Research (NIHR) recommendations [24]. Communication with the representatives was via email when sharing and receiving documents for review and opinion, and Microsoft Teams for discussion.

### 2.2 Study Phase 1

A cross‐sectional survey, co‐developed with our PPIE Stage 1 representatives, was employed to explore the experiences of individuals with larger bodies who had accessed UK radiography services (medical imaging and radiotherapy). Favourable University of Exeter (UoE) ethical approval was obtained (Ref: 1870378). Recruitment to the online survey was planned via social media platforms X (formerly Twitter), LinkedIn, TikTok and Facebook. This phase of the project and associated PPIE activity was funded by the College of Radiographers Industry Partnership Scheme (CoRIPS) (Ref: 232). The findings of the project have been reported separately [25].

### 2.3 PPIE Stage 2

Stage 2 of the strategy focused on the engagement of representatives to develop Study Phase 2 of the LBinRAD project. This stage was funded by the UoE Public Engagement with Research (PER) Springboard fund; these additional funds permitted the creation of a larger pool of representatives to help the researchers address the following objectives:

1. Explore the representatives' thoughts on appropriate (and inappropriate) terminology relating to the topic of larger bodies.
2. Explore with the representatives their thoughts on this topic in terms of the proposed research (i.e., does it seem of value?)
3. Explore how to maximise and widen participation in future research.
4. Co‐create statements of beliefs that would then be used to produce a Code of Conduct for any future research by this team on this topic.
5. Consider next steps (i.e., what might future research on this topic look like).

When compared to PPIE Stage 1 (to support Study Phase 1 of the project), the authors were mindful that these objectives would mean a sizeable increase in the volume of the activities which required representative input. Overburdening representatives with tasks could impact them negatively, hamper the progression of the project through the creation of bottlenecks, and result in the loss of any value which would be gained from PPIE activity due to superficial practices [26, 27, 28]. There was also recognition that our PPIE Stage 1 representatives, except for gender, lacked wider diversity, so it was imperative that PPIE Stage 2 was inclusive and provided stronger representation of the UK population.

To support recruitment, an LBinRAD website was built. This contained information on the project background, team members and details of existing project outputs (https://sites.exeter.ac.uk/lbinrad/). The site also housed a role descriptor (Appendix 1), detailing the different involvement opportunities available, estimated time commitment and the Honoraria to be paid alongside the Higher Education Institution's General Data Protection Regulation (GDPR) policies, including their right to have their personal details removed. A recruitment poster (Appendix 2) directed interested individuals to the website to find out more information and where they could sign up. Mirroring Stage 1, there was no target number of representatives. Recruitment included promotion on social media and across the research teams' own PPIE networks. The PPIE Stage 2 pool was also advertised through the NIHR's People in research site (https://www.peopleinresearch.org/). The four representatives involved in PPIE Stage 1 were also invited to continue contributing to Stage 2. Applicants were asked to provide, if they wished, a range of demographic and experiential details to help the researchers capture lived experience and expertise from a diverse group of individuals. To support the inclusive and non‐prescriptive nature of the research, the form enabled individuals to self‐identify or describe their gender, body shape, height and ethnicity. The details of those registering were captured on a Microsoft form; these were stored securely on Microsoft Excel in accordance with the GDPR. To facilitate PPIE integration and support the team to meet their objectives a series of online focus groups were planned. On invitation to the focus groups, the representatives were provided with the dates and session topics; commitment was again designed to be fluid to support representatives to engage where convenient and they felt they could contribute. An honorarium payment for attendance was offered, and if any pre‐reading was required, then payment was increased to reflect this additional time commitment.

## 3 Results

### 3.1 PPIE Stage 1

Four representatives expressed an interest to be involved and were invited to contribute their expertise to support and develop Study Phase 1 of the project. All four representatives were White, 2 identified as female and 2 as non‐binary, geographically they were in the Southeast (1), Central (2) and North (1) of England. All had previous experiences of medical imaging, but no representatives had experience with radiotherapy. Our PPIE strategy empowered the representatives to ‘cherry pick′ tasks which they felt comfortable and confident to undertake, that were of interest and could be completed at a time convenient to them. For all components of the research, there was active input from at least two representatives; involvement activities and the value which was added to the project are summarised in Table 1. To note, all four representatives refused the offer of payment.

**Table 1:** Stage 1: Involvement activities and outcomes.

Activities	Involvement and outcomes/Benefits
Research topic	*Project validation*
	Initial engagement activities highlighted personal insights of their own lived experiences of UK diagnostic imaging and radiotherapy services (both positive and negative), which reinforced the aims and objectives of the project.
Project acceptability	*Terminology*
	In‐depth discussions, which considered lived experiences and personal preferences on language and terminology, culminated in the collective agreement to use the term ‘larger bodies′.
	Supported by the agreement for the term to be used in combination with the explanatory statement ‘This includes anyone who is wider, taller or broader than the “average.” This includes but is not limited to those who are “plus size.′
	*Transparency*
	The challenges associated with participant labelling and bias, alongside any potential fear and mistrust of the research and its intent, led to the implementation of several key strategies (see survey design, participant‐facing materials and recruitment strategy sections below).
Survey design	*Data capture*
	Our PPIE representatives co‐designed the survey, providing insights on the range of clinical situations where having a larger body could influence experience (e.g., waiting areas, imaging/treatment areas, equipment use, and so forth), ensuring the data captured was reflective of real‐life diagnostic and radiotherapy clinical services.
	*Inclusivity*
	Our PPIE representatives provided their perspectives on the suitability of those self‐described demographic and personal characteristics requested and the wording of our non‐prescriptive requests. It was considered that this approach would make participants comfortable to disclose.
	Proofing and assessment of the survey (e.g., structure and terminology) were conducted in combination with piloting to enhance readability and promote accessibility across our target audience.
Participant‐facing materials	*Trust building*
	Our PPIE representatives actively co‐developed all outward‐facing recruitment materials, including the participant information sheets (PiS) and consent forms. Throughout these activities, our PPIE representatives addressed the necessity to be open and transparent about the intent of the research and the researchers.
	This resulted in:
	– The creation of the narrative overviews to provide the potential participants with genuine insights into the purpose of the research (survey). – Recommending the inclusion of potential reasons why the participants should take part to share their experiences, have their voices heard and impact change through helping to improve healthcare in recruitment materials.
	*Inclusivity*
	Imagery used on all materials depicted individuals with a range of protected characteristics (e.g., age, ethnicity and disability) and shapes and sizes to illustrate our ambition to be inclusive.
Ethical approval	*Quality enhancement*
	Integration of feedback gained from our representative on the proposed design and implementation contributed to favourable ethical approval.
	Lay summary co‐designed with PPIE representatives.
Funding application	*Quality enhancement*
	Integration of feedback gained from our representative on the proposed design and implementation contributed to the successful CoRIPs funding award.
	Continued consideration of the stage 1 feedback was also integrated within the application for PPIE stage 2 funding, and its implementation contributed to the successful PER funding award.
	Lay summary co‐designed with PPIE representatives.
Recruitment strategy	*Materials*
	Co‐developed all outward‐facing advertisement materials, throughout which our PPIE made recommendations to help us illustrate the transparency of the research and the researchers.
	These included:
	– Recommending any advertisement that contained photos and information on each member of the research team to humanise and help illustrate their motivations for undertaking the research. – Communication in social media posts outlining that numerical values for individuals' weight would not be asked for, as a way of reinforcing the message that this research was not ascribing a judgement to weight or had a subtext relating to optimal weight.
	*Widening participation*
	Whilst recognising that some people do not engage in social media and therefore the distribution strategy would favourably bias those that do, it was recommended by our PPIE representatives that using a wider spectrum of social media platforms than just X would help access a broader range and potentially more participants. The strategy was subsequently changed to include Facebook, Instagram and TikTok.
	*Gratuities*
	The researchers wished to appropriately compensate participants for their time. The online nature and anonymity of the survey, however, did not permit the reward to be directly provided to the participant; therefore, the team had originally considered using the funds to make charitable donations on the participant's behalf for every completed survey. Feedback provided from our PPIE representatives, however, illustrated the challenges of selecting a charity which all would be in favour of. It was also highlighted that amongst the larger‐bodied community, there was some negativity towards ‘weight′ associated charities due to the perceived pressure from these for individuals to lose weight to be considered *‘healthy′*. Based on this feedback, the decision was made to offer a donation on their behalf to a tree planting charity for the first 250 responses.
Data analysis and theme refinement	Opportunity for involvement in the analysis process was offered to the representatives; none of the individuals involved in PPIE Stage 1 accepted this offer.
Dissemination activities	Opportunity for involvement in the dissemination process was offered to the representatives; none of the individuals involved in PPIE Stage 1 accepted this offer.

### 3.2 PPIE Stage 2

28 new representatives were successfully recruited to the LBinRAD PPIE Stage 2 pool. None of the PPIE Stage 1 representatives committed to Stage 2, explaining changes in professional and personal circumstances no longer permitting them to continue as the reason. Each, however, wished to remain in contact and was open to potential involvement in the later study phases of the project.

### 3.3 Demographics

All participants described themselves as having a larger body and expressed that this was part of their motivation for joining. A range of demographic information was captured upon recruitment to help ensure diverse representation. In relation to radiography experience, 27 (96.4%) representatives had experience with medical imaging and 11 (39.3%) with radiotherapy, 10 of which (35.7%) will have experienced both. Self‐description of gender and/or sex established that 16 (57.1%) classified their gender as female, 1 (3.6%) as assigned female at birth (AFAB), 8 (28.6%) as male, 1 (3.6%) as cis‐male, 1 (3.6%) as non‐binary and 1 (3.6%) as straight. Representatives also had varied education as evidenced by their highest academic qualifications, and a range of ethnicities, regions and ages were represented (see Supplementary Information).

### 3.4 Focus Groups

With the consent of the representatives, all the focus groups were audio and video recorded to aid information capture and stored in accordance with the Higher Education Institute's GDPR policy. Each session was led by a facilitator and supported by a moderator, both of whom were members of the project team. A summary of the discussion was generated by the facilitator and verified by the moderator; however, to ensure each was a true reflection of the representatives' comments, the third team member reviewed the recording and contributed to the final summaries.

**Figure 3:**
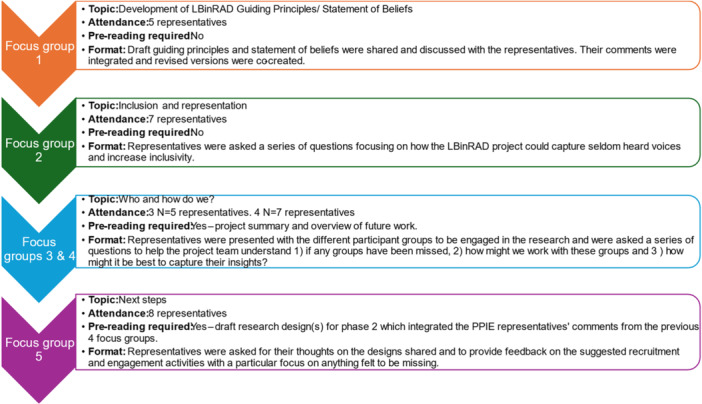
Summary of focus group attendance and format.

Each focus group started with brief introductions by the project team and representatives who were also asked to include their motivations for volunteering. The original format included a brief overview of the Study Phase 1; however, after the second focus group, this format was changed as the researchers found its inclusion restricted the time available to discuss focus session topics. Representatives were therefore provided with pre‐reading prior to Focus Groups 3, 4 and 5, which captured this information. The topic of discussion and format of each focus group are presented in Figure 3.

Twenty‐two members of the PPIE stage 2 pool attended across the five focus groups, all participants were provided payment for their attendance, and where prior reading was required, the attendees were paid for these preparatory activities. A document which captured a summary of key discussions and group consensus across the five sessions was devised and checked for lay appropriateness by a member of the LBinRAD PPIE stage 2 pool; this individual also received payment for this activity. The summary document was circulated to all the attendees as well as wider members of the PPIE stage 2 pool, serving as a process of member checking and an update on the project. Key learning and action points taken forward into Study Phase 2 of the project (and beyond) are provided within Table 2.

**Table 2:** Stage 2: Involvement learning and outcomes.

Summary	Key action points/Learning
Objective 1: Explore the representatives' thoughts on appropriate (and inappropriate) terminology relating to the topic of larger bodies.	
Language/Terminology	Continue to use the term ‘Larger Bodied′ but agreed for this to be reviewed for appropriateness and acceptability amongst PPIE representatives as an ongoing activity.
Self‐perception/description	Continue to provide the opportunity for PPIE/participants to self‐describe personal characteristics. Include a glossary of terms used and/or provide context for the use of particular words.
Objective 2: Explore with the representatives their thoughts on this topic in terms of the proposed research (i.e., does it seem of value?).	
Lived experiences	Provided validation of the aims and objectives of the LBinRAD project.
Objective 3: Explore how to maximise and widen participation in future research.	
Inclusion	Use of statement of beliefs (Objective 4) to create an environment which is safe and inclusive. Activities which support widening participation to be appropriately planned and costed as part of Stage 2. Lived experiences will be incorporated into future projects and funding applications, helping to provide the background narrative of the challenges faced by this group.
Recruitment	A range of approaches and advert locations is needed to ensure a range of participants. Widen the range of recruitment with advertisements targeted at specific groups. Building trust with local communities and networks to be appropriately planned and costed as part of PPIE Stage 3. Need for non‐digital options to be available for people who aren′t digitally connected. In‐person workshops might overcome this.
Imagery and wording/readability of recruitment materials	Simplifying language and reviewing materials with a lay audience as recommended. Need to consider language/sensitivities (Objective 1) when developing documentation (e.g., adverts, recruitment strategies and so forth) and co‐produce with those who have lived experience. Importance of messaging and transparency of the research and researchers. Use of alternative approaches (e.g., animations and videos). Using imagery to normalise people with larger bodies so that they are realistic and relatable, that is, by showing them doing everyday things as well as empowering activities (such as ‘*climbing mountains’*). Potential participants need to be able to see themselves in the images, and it's important that negative imagery/stereotypes are avoided.
Objective 4: Co‐create some statements of beliefs that would then be used to produce a Code of Conduct for any future research by this team on this topic.	
Statements of beliefs	The following statements of beliefs were agreed upon by the representatives in attendance:
	1. We believe in treating each other with dignity, respect and professionalism. This includes using inclusive and transparent language, avoiding criticism of body size/avoiding weight‐shaming. 2. We believe in fostering a supportive community where everyone feels safe, valued and empowered. 3. We believe in separating appearance from health. Each body is different, and we reject medical biases that equate health with weight. 4. We encourage healthcare and social care providers to focus on holistic well‐being rather than solely on body size.
Objective 5. Consider the next steps, that is, what future research on this topic might look like.	
Populations	Recommendations to gain insights from people with larger bodies, healthcare professionals, manufacturers, students and academics.
Methods	1:1 conversation/interviews Focus Groups Online forums
Topics/focus	Academic teaching/student training Clinical practices Clinical equipment/Industry/manufacturers Mental health Integrated into the development of Study Phase 2 of the research and beyond.

## 4 Discussion

Guided by the rationale for the research and a recognition of those longstanding sensitivities surrounding the topic of weight/size meant it was of paramount importance that inclusive language, terminology and messaging were considered to support our initial PPIE recruitment strategy. As the project progressed, the complexities surrounding the topic and those individuals under the research lens continued to unfold. Our robust yet flexible strategy enabled the team to meaningfully engage with our representatives, addressing potential barriers to the research by adapting central elements of recruitment strategy and methodological design in Study Phase 1 (Table 1). Employment of a poorly considered PPIE approach or strategy can be detrimental to the success of the research, resulting in a loss of opportunity for inclusive practice [29, 30]. The triumph or indeed the failure of this research lay with the successful recruitment of PPIE representatives across every phase of the project, inclusive of those individuals who perceived themselves to be reflective of the larger‐bodied population. Without this appropriate representation, the voices of those key individuals under the research lens would be lost [29, 30].

Getting the language ‘right′ from the start was critical; affronting our target population would have caused alienation and a loss of trust from those our research was seeking to benefit. And whilst our PPIE illustrated it was challenging to find encompassing terms that were agreeable with everyone, ‘Larger body′ was perceived as appropriate as it was felt to be a softer term, especially when used in a neutral way (i.e., a person with a larger body). Although Study Phase 2 at the time of this publication is in development, the successful participant recruitment to Study Phase 1 would indicate that the language/terminology used did not offend or alienate survey participants. Similarly, the recruitment and continued support of our pool of PPIE representatives illustrates how our messaging has been effectively shaped to clearly articulate our intent, enabling us to create a community of respectful engagement [31, 32].

### 4.1 Shaping Outcomes: The Contribution of PPIE

#### 4.1.1 Validating Lived Experiences

Personal experiences within the PPIE groups validated how larger‐bodied individuals can have negative experiences of healthcare. There was a strong alignment between our representatives' perspectives and research that has observed how healthcare professionals do not always see the whole person, instead focusing on someone's body shape/size (or healthcare condition), potentially seeing that as the only source of any health‐related issues. This subsequently can become a barrier to person‐centred care [33]. Our representatives felt strongly that healthcare practitioners need to remember that not everything is about body size, but also stressed how this could also apply to anyone perceived by the healthcare system as ‘non‐standard′ (such as people with dementia or autism). There was a consensus that, regardless of individual characteristics, everyone should receive person‐centred care, respected and treated inclusively by professionals who use non‐judgemental language [34, 35].

#### 4.1.2 Widening Participation

A prominent and consistent message across all PPIE activity was the importance of creating a welcoming and inclusive environment, where a diverse group of people who regard themselves as having a larger body would be comfortable to attend and contribute [36, 37, 38]. Continuing to outwardly reflect our research intent (shortcomings with technology and healthcare and not the individuals living in larger bodies) whilst also sensitively balancing the use of terms which may be acceptable to some (e.g., fat for those in the body positive sphere) whilst offensive to others, underpinned the rationale for developing a statement of beliefs. Like ground rules, co‐designed statements of beliefs foster environments where individuals and their differences, including opinions and beliefs, as well as demographics, are mutually respected [39]. These co‐designed statements will provide the foundation of all future LBinRAD PPIE and research activities, outlining the necessity to respect and be respectful of the differences amongst the group. This will be especially important during the latter stages of the research, where different cohorts of participants (professional, academic, manufacturers and so forth) will be collectively engaged in co‐production/co‐design [30, 40].

Moving forward into the research Study Phase 2, participation will extend to those who may not necessarily be living in a larger body right now, as those who have had a larger body in the past may also have valuable experiences and insights to share. Accessing gyms/sports clubs or weight loss clubs will be adopted as a method to increase the reach of the recruitment, allowing the research to consider the ‘range′ and fluctuation of weight and shape [41]. Similarly, we will address the need to engage with a broader range of people and especially those who may feel less inclined to participate in research due to societal pressures and/or feeling stigmatised [36, 42]. A range of approaches have been previously adopted, including the utilisation of trusted community leaders, organisers, established groups and charities as a way of reaching specific communities, including those with disabilities [43, 44, 45], thus helping to build trust and supporting recruitment from seldom heard voices or communities [26, 36]. Wider inclusivity measures, which can enhance participant recruitment, including translators and translation of key study documents for those with limited English Proficiency [46], Makaton/British sign language to provide access to deaf individuals and those who are hard of hearing [47], will be adopted in conjunction with promoting research opportunities wider than social media to increase representation [48].

Our PPIE suggests the use of recruitment materials and methods of qualitative data collection which move away from traditional formats (e.g., posters and structured questions) towards more creative methods (e.g., cartoons and comedy). It was, however, recognised that any use of humour must be well balanced to avoid the potential of someone with a larger body feeling they are the target of the joke. Similarly, it was stressed how we must also consider cultural sensitivities, and the relationship people from specific cultures may have with healthcare providers and researchers [44] as well as body composition (weight/shape/size and so forth) [49]. Different messaging may be needed for different cultural/social groups to reflect understanding of their unique perspectives, and language also needs to be culturally inclusive. Our PPIE activities also highlighted how the language used across our methodological approach needs to portray patients as respected collaborators rather than ‘victims′ to encourage participation and honest feedback [31].

### 4.2 Lessons Learnt

Our work has presented the opportunity for reflection and the collation of ‘lessons learnt′ which will support the continuation of this and future projects.

#### 4.2.1 PPIE Recruitment

Sensitivity and diplomacy are essential when PPIE recruitment is purposefully aimed at individuals with a specific demographic or characteristic. This is particularly important when trying to engage those who may have experienced previous societal or research‐based discrimination, judgement or ridicule due to a sense of mistrust [42]. Subsequently, consideration should be given to PPIE strategies to support the recruitment of research participants, stopping the exclusion of specific population groups [29].

#### 4.2.2 Reinforcing Negative Views

Within our research, simply approaching individuals to contribute their perspective to the project could have reflected a judgement based on how that person looks, potentially illustrating that we as a research team perceived them to be ‘larger bodied′ and subsequently eligible to participate. As illustrated, there may be a conflict with how people view themselves compared to how the external world views them. It is therefore essential when recruiting to PPIE positions that researchers are respectful in their approach, leaving the decision of eligibility to the individual rather than approaching them based on their physical characteristics.

In future, the collation and presentation of demographics also need to move away from a ‘one size fits all′ method, which has traditionally required individuals to fit themselves into a predefined box. Using a flexible approach empowers individuals to self‐describe, eliminating the use of pre‐assigned labels and promoting research inclusivity [50].

#### 4.2.3 Disparate Preferences

Incongruent acceptability of the term ‘fat′ was evident between the PPIE representatives, this was particularly demonstrated in the second focus group. Whilst the term had been used openly by some to self‐describe, founded on their belief that the term had been reclaimed as part of the body positivity movement, others found the term emotionally triggering. Researchers must recognise that the way someone describes their own characteristics is personal to them; subsequently, not all terminology will be acceptable or agreeable to all and may even be rejected [51]. Research that focuses on sensitive and emotive topics should therefore continue to address the appropriateness of terminology by engaging PPIE representatives.

### 4.3 Strengths and Limitations

As a project group, we recognise that we are not representative of those individuals whom we wish to engage as research participants; instead, we are healthcare professionals working in research and academia. Through the participants' lens, we may be judged as those who have alienated them from healthcare services and are part of the problems which they face, rather than part of the solution. Our employment of a strong, flexible and inclusive PPIE strategy was intended to overcome this limitation, strengthening the research whilst ensuring the patient voice is central and reflective of those it seeks to improve healthcare experience for.

Whilst a conscious attempt was made to enhance the diversity across our representatives, we acknowledge how our PPIE pool may not have fully captured those individuals who reflect the wider society or our future participant demographic. Furthermore, we recognise that by only having four PPIE representatives to support Stage 1 of the PPIE strategy, there may be areas of Study Phase 1 that did not benefit from a broader range of lived experiences. Recruitment to our PPIE pool will be ongoing throughout the lifespan of the LBinRAD project and will utilise those suggestions made to widen participation and promote inclusivity for Study Phase 2 into this process. A limitation of reporting PPIE activities is that it does not follow formal research recruitment practices, data collection or analysis. Attempts to formalise the process can push these activities into research, fundamentally changing their purpose. This leads to challenges with both reporting and dissemination; both activities can help with the development of good PPIE practice. Within our work, attempts have been made to strengthen the robustness of PPIE activities, such as adherence to the INVOLVE and NIHR guidance [22, 23, 24].

## 5 Conclusion

As the number of individuals with larger bodies accessing radiography services is increasing, it is imperative that research is undertaken to support the development and implementation of clinical environments and those healthcare professionals who practice within them to be inclusive, non‐judgemental and person‐centred. This is also needed to address inequalities in healthcare, such as those associated with weight stigma, delayed diagnosis due to challenges accessing diagnostics, and/or healthcare avoidance. Employment of our PPIE strategy has brought success to our Study Phase 1 in what has unfolded to be a sensitive and complex field, helping us to understand the current real‐life challenges faced by those with a larger body. Our experiences have reinforced for us, as researchers, the value of a flexible, longitudinal approach to the PPIE activities themselves, to maximise participation, but also in recognition of the fact that opinions can and will vary amongst individuals even when a particular characteristic is shared. Our PPIE representatives have also provided valuable insights about key aspects which will be key to the development of Study Phase 2 and ensure that this, as well as our ongoing PPIE activities, are guided by our statement of beliefs.

## Author Contributions

**Amy Hancock:** conceptualisation, investigation, funding acquisition, writing – original draft, methodology, validation, visualisation, writing – review and editing, project administration, formal analysis, software, data curation, supervision, resources. **Christine Heales:** conceptualisation, investigation, funding acquisition, writing – original draft, writing – review and editing, visualisation, validation, methodology, software, formal analysis, project administration, resources, supervision, data curation. **Poppy Ulett:** methodology, validation, formal analysis. **Graham Carolyn:** methodology, validation, writing – review and editing, conceptualisation. **Fay Manning:** conceptualisation, investigation, funding acquisition, writing – original draft, writing – review and editing, visualisation, validation, methodology, software, formal analysis, project administration, data curation, supervision, resources.

## Ethics Statement

Ethical approval was obtained for the Study Phase 1 of this research from the University of Exeter (Ref: 1870378). PPIE data was obtained and stored in accordance with the University of Exeter General Data Protection Regulation (GDPR) policies.

## Consent

Informed consent was obtained from all participants.

## Conflicts of Interest

The authors declare no conflicts of interest.

## AI Statement

No generative AI or AI‐assisted technologies have been used in the writing of this paper.

## Supporting information

Appendix 1_ PPIE role descriptor.

Appendix 2 LBinRAD Bank PPIE Poster.

Supplementary information. PPIE Pool demographics.

## Data Availability

The data that support the findings of this study are available on request from the corresponding author. The data are not publicly available due to privacy or ethical restrictions.
