# Supplementary material for: Researching Sensitive Topics: The Value of Inclusive Patient and Public Involvement and Engagement in the Design and Implementation of the Larger Bodies in Radiography Project
Source: Health Expect. 2026 Feb 8;29(1):e70563. doi: 10.1111/hex.70563 (PMC12883691; doi:10.1111/hex.70563)
Supplement: Supplementary file 1 — Appendix 1_ PPIE role descriptor. [file HEX-29-e70563-s002.pdf]

Larger Bodies in Radiography (LBinRAD)

PPIE Bank Recruitment

TYPE OF OPPORTUNITY	TIME COMMITMENT	HONORARIA
Public/Patient (PPIE) Bank Member	Flexible	£25 p/hr for meetings and material reading/prep.

STUDY BACKGROUND

There is evidence that people who have larger bodies (this includes anyone who is wider, taller or broader than the 'average' and is not limited to those who are 'plus size' may have negative experiences when accessing healthcare services. Both the person themselves, and the healthcare professional, may for example encounter challenges due to limitations in the design of radiographic equipment because of weight and/or size. Current research on this topic tends to focus on these more technical aspects and until this project, there has been little information available about the experience of people with larger bodies who are referred to radiography services.

We therefore undertook a survey that aimed to ask people living in larger bodies what their experiences of accessing radiography services (either Medical Imaging or Radiotherapy) have been. This included questions on attitudes and behaviours of the staff, and their own experiences, as well as about resources and equipment. Unsurprisingly, survey participants expressed a range of experiences, many negative leading to ongoing consequences outside of the clinical visit.

We are therefore planning to use the results of this survey to develop a follow-on project to explore key areas in more detail. The longer-term aim would be to co-create resources for patients, clinical radiographers and healthcare educators about larger bodies in radiography services, so we can provide the best possible care for this group of people.

You can see a bit about our study on our social media platforms:

- X/Twitter: @LBinRAD
- Instagram: @lbinrad
- Facebook: LargerBodies InRadiography
- Website: https://sites.exeter.ac.uk/lbinrad/

OPPORTUNITY SUMMARY

To inform the development of the next study, we are recruiting a ‘PPIE Bank’ (a bit like a mailing list) of people who are willing to contribute in a variety of ways. This would involve providing a small amount of information on yourself (name, age, ethnicity etc), contact details and permission for us to hold these details and contact you.

When an opportunity for input appears, we will email the PPIE Bank asking for interest in that particular task. We would include details on what to expect/do, how long we would expect the task to take, and when it would occur/a deadline. We would either offer the task on a first come first served basis or purposively choose certain people from those interested based on specific criteria to ensure diversity of views.

Type of tasks could include (but are not limited to) taking part in workshops/focus groups to develop aims and methods, reviewing documents to check clarity and understanding and giving feedback on images used in public-facing materials. The majority of tasks will be digital (e.g. word processing) or online (e.g. Teams or Zoom).

It would be completely your choice what activities to participate in, feel free to play to your strengths or try something new! Not everyone would be expected to take part in all activities, and there is no minimum level of involvement to stay on the PPIE Bank list.

TIME OF COMMITMENT

At present, the PPIE Bank is likely to exist for around 2 years, but depending on project funding this may extend. You would be able to stay in the PPIE Bank for as long as you wish, likewise, it would be completely up to you if you would like to withdraw from the Bank prior to its end date. Once you have joined the PPIE Bank, if you would like us to stop contacting you and delete your information, please contact Fay Manning (f.manning2@exeter.ac.uk).

The time commitment for each task may vary. As previously described, each optional task offered to the PPIE Bank will come with details on what to expect/do, how long we would expect it to take, and when it would occur/a deadline. Some tasks may be short, i.e. commenting your opinion on an image, and others may involve multiple steps e.g. reading through documents and then joining a workshop to discuss them, and reviewing an updated version. Your involvement and time commitment levels will be completely up to you.

COMPENSATION FOR YOUR TIME

Honoraria (a voluntary fee for a voluntary service) will be given for time taken to complete tasks at the rate of £25 p/hour. We do not offer any pay for being listed on the PPIE Bank.

Each task will have an expected duration attached, we would ask that you communicate with us prior to spending substantially longer on a task than outlined so that we may budget accordingly.

To be paid, you will be asked to provide bank details. These will be held in password-protected encrypted files only accessible by the research team, and only shared with the University of Exeter Finance department for the purpose of fund transfer (see data protection section below for more details).

You may wave the honorarium, however, we are unable to donate it to charities on your behalf.

SUMMARY

YOUR SKILLS AND EXPERIENCES	YOUR ROLE
We are looking for people who…<br>- Are aged 18 years of age or over<br>- Identify as having a 'larger body'<br>- Have experience of the UK health services (private or NHS)<br>- Can provide critical and constructive feedback<br>- Have basic IT e.g. use of Microsoft programmes and zoom<br>- Have confidence to voice their own opinions and participate in group discussion<br>- Are able to listen and respect differing opinions<br>- Are aware of equality and diversity<br><br>You <u>do not</u> need to have…<br>- Experience of Medical Imaging or Radiotherapy<br>- Any previous experience of research	You will be expected to …<br>- Respect the viewpoints of others in the group<br><br>You will have the options to …<br>- Attend and take an active part in online workshops and focus groups<br>- Review documentation, marketing and imagery<br>- Share your opinions and give advice<br>- Ensure that discussions consider how the work fits with our research strategy and is helping to drive patient benefit<br>- Provide a lay perspective on operational and strategic issues<br>- Take part in dissemination (getting the word out)

HOW TO SIGN UP

- Complete the Microsoft Forms: https://forms.office.com/e/NKkwHFzQDs
- For any questions, contact LBinRAD PPIE Lead Dr Fay manning – f.manning2@exeter.ac.uk

DATA PROTECTION AND GDPR

The University of Exeter (the University) is a data controller and will only process your personal data in accordance with the University's data protection notice and in accordance with the current Data Protection
legislation (which includes the Data Protection Act 1998 or, from the date it comes into force, the General Data Protection Regulation). Details of the University's Data Protection Officer can be found at http://www.exeter.ac.uk/dpo/
Research teams will only keep your personal data for as long as necessary, bearing in mind the purposes for which your personal data is collected, and will explain clearly how long your data will be kept for each project. You can withdraw your data at any time by emailing Fay Manning (f.manning2@exeter.ac.uk). Further general information on how long the University will keep data is also available on the University Retention Schedule.
You have a number of rights in relation to your personal data. Information about what these rights are and how to exercise them is available at www.exeter.ac.uk/dataprotection/rights.

If you have any concerns about your data you can contact the data protection officer:
Email: informationgovernance@exeter.ac.uk
Tel: 01392 726842

ADDITIONAL BACKGROUND INFORMATION

Due to the topics being discussed, respect and confidentiality for other PPIE Bank members is paramount. As such part of our initial activities will involve developing 'community guidelines' that all contributing will be asked to agree to. These will aim to foster inclusion, safety, trust and respect within all our activities.

FUNDING

The initial LBinRAD Survey study was funded by The College of Radiographers Industry Partnership Scheme (CoRIPS). This patient and public work is funded by the University of Exeter Public Engagement with Research (PER) Springboard Fund.

CONTACTS

Study Lead: Dr Amy Hancock, a.hancock@exeter.ac.uk

PPIE Lead: Dr Fay Manning, f.manning2@exeter.ac.uk
