# Supplementary material for: Researching Sensitive Topics: The Value of Inclusive Patient and Public Involvement and Engagement in the Design and Implementation of the Larger Bodies in Radiography Project
Source: Health Expect. 2026 Feb 8;29(1):e70563. doi: 10.1111/hex.70563 (PMC12883691; doi:10.1111/hex.70563)
Supplement: Supplementary file 2 — Appendix 2 LBinRAD Bank PPIE Poster. [file HEX-29-e70563-s003.pdf]

We are recruiting for the Larger Bodies in Radiography (LBinRAD) Patient and Public Involvement and Engagement Team!

Join now

If you identify as having a larger body (height and weight inclusive) and have experience of UK health care we want to hear from you!

Find out more and sign up HERE

University of Exeter
