# Supplementary material for: Researching Sensitive Topics: The Value of Inclusive Patient and Public Involvement and Engagement in the Design and Implementation of the Larger Bodies in Radiography Project
Source: Health Expect. 2026 Feb 8;29(1):e70563. doi: 10.1111/hex.70563 (PMC12883691; doi:10.1111/hex.70563)
Supplement: Supplementary file 3 — Supplementary information. PPIE Pool demographics. [file HEX-29-e70563-s001.docx]

Supplementary information: PPIE Pool demographics

| Region | North West = 4  South West =4  South East =4  East of England =2  North East =2  West Midlands =1  East Midlands =1  Scotland =2  Northern Ireland N=1  North East =1  London =5  Wales =1 |
| --- | --- |
| Highest level of education | Doctorate (PhD), Level 8 awards, diplomas and certificates or equivalent = 1  Masters degree, Level 7 NVQ, diplomas or certificates, Postgraduate diplomas and certificates or equivalent =7  Bachelors degree, Level 6 NVQ, diplomas or certificates, degree apprenticeships or equivalent = 10  A level, Level 3 NVQ, International Baccalaureate diploma or equivalent = 4  GCSE, O level, Level 1 & 2 NVQ or equivalent = 3  Level 4 and 5 NVQ, diplomas or certificates, CertHE, DipHE or equivalent = 2  Did not wish to disclose = 1 |
| Ethnicity | White British = 10,  White = 2  British = 1  Black British = 3  Asian British Indian = 1  South Asian = 1  Asian = 2  White Asian = 1  Asian Pakistani British =1  Bangladeshi =1  Mixed White/Asian =1  Welsh =1  White European =1  Indian =1  Prefer not to say =1 |
| Age ranges | 18 – 25 =2  26 – 35 =8  36 – 45 =5  46 – 55 =9  56 – 65 =3  76 – 85 =1 |
